# Follow-up descriptive study of how proportioning marks between coursework and examination affects the performance of students in nursing

**DOI:** 10.1186/s12912-023-01286-w

**Published:** 2023-04-25

**Authors:** Sheila A Doggrell

**Affiliations:** School of Pharmacy and Medical Sciences, Griffith University, Gold Coast Campus, Queensland, Australia

**Keywords:** Bioscience, Examination, Individual laboratory skills, Marks, Nursing students, Coursework, Team assignment/communication

## Abstract

**Background:**

There has been little attention to how the allocation of marks affects the academic performance of students in courses. Our previous study showed that students in nursing had much lower marks in exams than coursework (tutorials and case study) in a pharmacology course. It is not known whether this applies to nursing students in other courses and/or with different types of coursework. The purpose of this study was to analyse how the allocation of marks to examination and different coursework affected the performance of students in nursing in a bioscience course.

**Methods:**

For the 379 completing students in a nursing degree undertaking a first-year first semester bioscience course, a descriptive study was undertaken of (i) the marks for the exam and two coursework components (individually undertaken laboratory skills, and a team/group project on health communication), with the marks being compared by Students t-test, (ii) any association between these marks was determined by regression line analysis, and (iii) modelling was undertaken to determine the effects of changing the allocation of marks on passing and failing rates.

**Results:**

Students in nursing who completed a bioscience course had much lower marks in the exam than the coursework. Regression line analysis of the marks in the exam versus combined coursework showed (a) a poor line fit and (b) the correlation coefficient was moderate (r = 0.51), for the individual laboratory skills vs. exam was moderate (r = 0.49), but only weak for the group project on health communication vs. exam (r = 0.25). A high percentage of students passed the course (97%). Modelling showed that increasing the marks for the exam decreased the number of students passing the course to as few as 57%.

**Conclusions:**

The allocation of marks determines the percentage of students in nursing who pass courses, regardless of the type of coursework. The students in nursing in the bioscience course, who pass the course based on marks from coursework, but not the examination component, may not have the necessary knowledge to continue their program of study. Thus, requiring students in nursing to pass exams should be given further consideration.

## Introduction

Most courses are a mixture of exams and coursework. Exams test the taking in and understanding of knowledge while ensuring that the students undertake the work themselves [1]. Performing well in exams is especially important for students in nursing in some countries as it determines whether they can practice clinically. For instance, in the USA and Canada, after completing university studies students must pass national exams before they can practice nursing. This contrast with the UK, the Republic of Ireland, Australia, and New Zealand, which do not require nursing students to pass a national exam prior to practising nursing but require graduation from an accredited course with exams and coursework.

Assessment can either be summative, which evaluates student learning at the end of the component or course, or formative, which is ongoing and monitors students learning to provide feedback to the student and teacher. Whereas examinations are clearly summative, coursework can be either summative or formative. One of the reasons for this is that coursework takes many forms including weekly quizzes, homework, games, tutorials, laboratory work, oral or poster presentations, and assignments/research projects [2]. In addition, coursework can be individual or group activities. Some of these coursework types are examples of formative activities e.g., weekly quizzes and homework, whereas others are summative e.g., final presentations and final reports [3]. An additional complication is that quizzes and tests overlap and may be formative or summative. Thus, weekly unmarked quizzes are clearly formative, whereas marked quizzes/tests that ‘exam’ a component of the course are probably summative.

In programs, there is evidence that the marks for coursework are higher than for the exams undertaken at the end of the individual course, and therefore, students have higher overall marks in programs with more marks for coursework, and consequently better degrees [4–6]. This may also apply to students in single courses, with students in the biology/molecular sciences having higher marks in courses with only coursework assessment, compared to courses with mixed assessment [7]. In these studies, the reason why students have higher marks in coursework than examinations was not elucidated.

The studies of the relationship between marked coursework and marks in exam has not been studied for students in science and there are only a few studies for students in allied health. The two studies in allied health courses have shown only a weak correlation between the marks for coursework and exams for pharmacy students [8] and for students in nursing in a pharmacology course [9]. In the pharmacy program, it was shown that in most of the courses, coursework marks were clustered between 60 and 80% and were higher than the exam marks [8]. In the study of students in nursing undertaking a pharmacology course, it was shown the students had much lower marks in the pharmacology exams than the coursework. This was true for the overall coursework and each of the two components: weekly tutorials with marks for individual and group components, and an individually undertaken written case study [9]. My theory was that marks for exams in other disciplines undertaken by students in nursing would also be higher for coursework than exams, regardless of the type of coursework. In the bioscience course studied in the present, the coursework was individual laboratory skills and a group project on communications in health, which was different than previously studied.

In most countries and within most countries/universities, there are no rules about the proportional allocation of marks between coursework and exams, and the allocation is often made on a seemingly arbitrary basis and not justified. Bioscience courses are taught as part of undergraduate BNursing degrees in Australia. At Queensland University of Technology (QUT) the first year, first-semester bioscience course (Anatomy and Physiology for Health Professionals) is a pre-requisite for two further courses in the nursing program: first-year, second semester Pathophysiology for Health Professionals, second year, first-semester Introduction of Clinical Therapeutics for Health. In the first-semester bioscience course at QUT, 60% of marks are allocated to the coursework and 40% to the theory exam at the end of the course. In other bioscience courses for nurses in Australia, the proportion of marks allocated for exams including marked summative quizzes/tests ranged from 15 to 100% in 2022 with the remainder of the marks allocated to coursework. Thus, the allocation to exams was 15% at RMIT University [10], 35% at Edith Cowan University [11], 40% at Monash University [12], 60% at University of Tasmania [13], 90% at The University of Queensland [14], and 100% at Charles Darwin University [15].

At some universities, the proportional allocation of marks may have been changed in the Covid-19 pandemic due to the difficulty of running internal exams. The consequences of the proportional allocation of marks in courses are often not considered. We have recently shown that in a pharmacology course, if the proportion of marks allocated to exams had been increased, this would have increased the failure rates for students in nursing [6]. It is not known whether these findings of low marks in exams and a weak relationship between coursework and exams apply to other courses for nursing or to other types of coursework.

The present study was of the marks obtained by students in nursing studying a first-year course in bioscience (Anatomy and Physiology for Health Professionals) to test the following hypothesise.

(i) That nursing students in bioscience would have higher marks in coursework than in the exam.
(ii) The hypothesis was that, regardless of the type of coursework, these marks would predict marks in the exam.
(iii) That allocating more marks to the exam would be associated with lower overall marks and pass rates.

## Methods

This study involved students in the first year of a nursing degree undertaking a bioscience (anatomy and physiology) course at QUT.

### Study design

The design is a descriptive study of the relationship between mark allocation to an exam and coursework (laboratory skills and communications in health) and the academic outcomes for nursing students in a bioscience course.

### Research setting

The research was undertaken in an Australian university, where students are typically required to achieve an overall mark of 50% to pass a course and passing grades are 4 (overall mark, 50–64%), 5 (65–74%), 6 (75–84%) and 7 (≥ 85%). Thus, in the bioscience course, students with < 50% of the overall marks failed the course.

### Course details

In the bioscience course, the learning outcomes were.

(i) Explain the complementary relationships that exist between tissue/organ structure and the functions of each of the major organ systems than contribute to homeostasis and the maintenance of life.
(ii) Accurately identify key anatomical structures through visual inspection, and analyses and interpret physiological data.
(iii) Effective communicate and share knowledge of anatomy and physiology concepts in a collaborative health care context.

In the course, 40% of the mark was allocated to the final (theory) exam, which examined the understanding of foundational concepts in anatomy and physiology and was mostly based on lecture content (2 h/week over 12 weeks) and was considering the first and second learning outcomes. The final theory exam had 65 MCQs and 5 SAQs (5 marks each). The other 60% of the total marks were allocated to coursework, which had two components: laboratory skills (35%) and communications in health (25%). The laboratory skills required individual students to identify anatomical structures on anatomical models, and to describe functions. For the laboratory skills training, microscopes/histological sections, anatomical models, embalmed human organs, and fresh animal tissues were used. The laboratory skills address the first and second learning outcome. For the communication in health coursework, the students had to work in teams to create a digital resource on a clinical case suitable for an audience of health professionals describing anatomy and physiology in a healthcare environment and using medical terminology. This communication in health coursework addresses the third learning outcome.

### Participants

The bioscience course is undertaken by students in their first year at university, and most of the students have recently completed secondary/school education. In semester 1 2019, the course had 389 students in nursing enrolled initially. Most of these were enrolled in a BNursing programme (261 students) with the remainder enrolled in Joint BNursing programmes with BParamedicSc (75 students), BBehavSc(Psych) (47 students) or BPubHealth programs. Some of these withdrew early (before census date), so that at the end of the course, 379 nursing students completed, with 10 of these students failing, seven of whom did not sit the final exam.

### Data collection procedures

The author was not involved in the bioscience course. The coordinator of the course gave their permission for the author to undertake the study and provided the author with a copy of the Microsoft Excel sheets of the marks associated with the course. This data was starting point for the following analysis. In the analysis, P ≤ 0.05 were considered significant for both Student’s t-test and Odds ratios.

The methods for data analysis used in this study have previously been published by Doggrell [9, 16].

*Data analysis for comparing academic perform*ance *in coursework and the exam as previously described* [9].

The marks for the combined coursework (laboratory skills and communications in health), the components of the coursework, and the exam were totalled, the total expressed as a percentage, and then the percentages were averaged. The percentages for individuals in the exam, overall coursework, and the components of coursework were compared by Students paired t-test. Mean values ± SD were also determined.

*Data analysis for regression line analysis for the passing students to determine whether performance in coursework was a predictor of performance in the exam* [9].

To determine Pearson’s correlation and significance, regression line analysis was undertaken using the data analysis function in Microsoft Excel. Coefficients of 0–0.19 were considered very weak, 0.2–0.39 weak, 0.4–0.59 moderate, 0.6–0.79 strong, 0.8–1.0 very strong: http://www.statstutor.ac.uk/resources/uploaded/pearsons.pdf. The marks for individual students in the exam were also plotted against their marks in the combined coursework and the components (laboratory skills and communications in health). The equation for the regression line (y = ax + b), where ‘a’ is the slope of the line, and the R^2^ values were also given. In regression, the R^2^ coefficient of determination is a statistical measure of how well the regression line approximates the real data points, with an R^2^ of 1 indicating the regression line perfectly fits the data.

For all the students who completed the course (i.e., successful and failing students), modelling was undertaken to determine the effect of changing the marking proportions from 40% combined coursework/60% exam had on the pass/failure rates and overall grades. The proportions modelled were changed to (i) 60% for combined coursework and 40% for exam, (ii) 80% coursework /20% exam, (iii) 100% coursework /0% exam, (iv) 20% coursework /80% exam and (v) 0% coursework /100% exam. Mean values ± SD were determined. Students who achieved less than 50% in the combined coursework or the exam were considered to have failed that component for both the actual and modelled data.

*Data analysis for how proportioning marks, between coursework and the exam, affected the overall marks and pass rates for the passing and failing students* [8].

Students who achieved less than 50% in the coursework or the exam were considered to have failed that component; failure rates for each component were compared by Odds ratio using the online Odds ratio calculator; https://www.medcalc.org/calc/odds_ratio.php.

## Results

The results are for the 379 completing students, who had a passing rate of 97.4% and the failure rate of 2.6%.

### Comparison of marks for the exam and coursework

Students obtained significantly lower marks, 23%-point difference, in the exam than coursework (Table 1). Dividing the coursework showed that students obtained significantly lower marks, 4%-point difference, in the group project communication in health than in the individual laboratory skills (Table 2).

**Table 1:** Percentage marks (left) and failure rates (right) in the exam and coursework

Percentage marks			Failure rates		
Exam	Coursework	Paired t-test	Exam	Coursework	Odds ratio, P value
51% ± 18	84% ± 9	P < 0.0001	164/379 (43.3%)	6/379 (1.6%)	27.3, P < 0.0001
Laboratories	Communication		Laboratories	Communication	
86% ± 12 (379)	82% ± 12 (379)	P < 0.0001	3/379 (0.8%)	3/379 (0.8%)	1.0, P = 1.0
Exam vs. laboratories		P < 0.0001	Exam vs. laboratories		54.7, P < 0.0001
Exams vs. communication		P < 0.0001	Exams vs. communication		54.7, P < 0.0001

Each value is the mean from 379 students ± SD. Failure rates were number of student with less than 50%/total number of students who passed the unit (percentages). Paired t-tests are between marks, and odds ratio are between failure rates.

**Table 2:** Values from linear regression of the exam vs. coursework

	(n)	Slopes	R^2^	r	Significance
Exam vs. coursework (combined)					
Completing	379	0.9981	0.261	0.51	P < 0.0001
Minus students who did not sit exam	372	0.8909	0.218	0.47	P < 0.0001
**Exam vs. laboratory skills**					
*Completing*	379	0.7287	0.240	0.49	P < 0.0001
Minus students who did not sit exam	372	0.6489	0.200	0.45	P < 0.0001
Minus students who did not sit exam or undertake laboratories	369	0.7227	0.210	0.46	P < 0.0001
**Exam vs. communication in health**					
Completing	379	0.4226	0.074	0.25	P < 0.0001
Minus students who did not sit exam	372	0.3332	0.052	0.23	P < 0.0001
Minus students who did not sit exam or undertake laboratories	369	0.3524	0.034	0.19	P = 0.0004

r = Pearson’s correlation coefficient

Despite passing the bioscience course overall by obtaining ≥ 50% of the total marks available, some of these students failed the individual components, by obtaining < 50%. Thus, the failure rate for the exam was 43.3%, which was much higher than for the coursework; 1.6% (Table 2). Of the six students who failed a component of the coursework, three failed the laboratory skills with one student not undertaking this component, and all three students who failed the communication in health did not undertake this component.

### Regression analysis and Pearson’s correlation coefficients

**Fig. 1:**
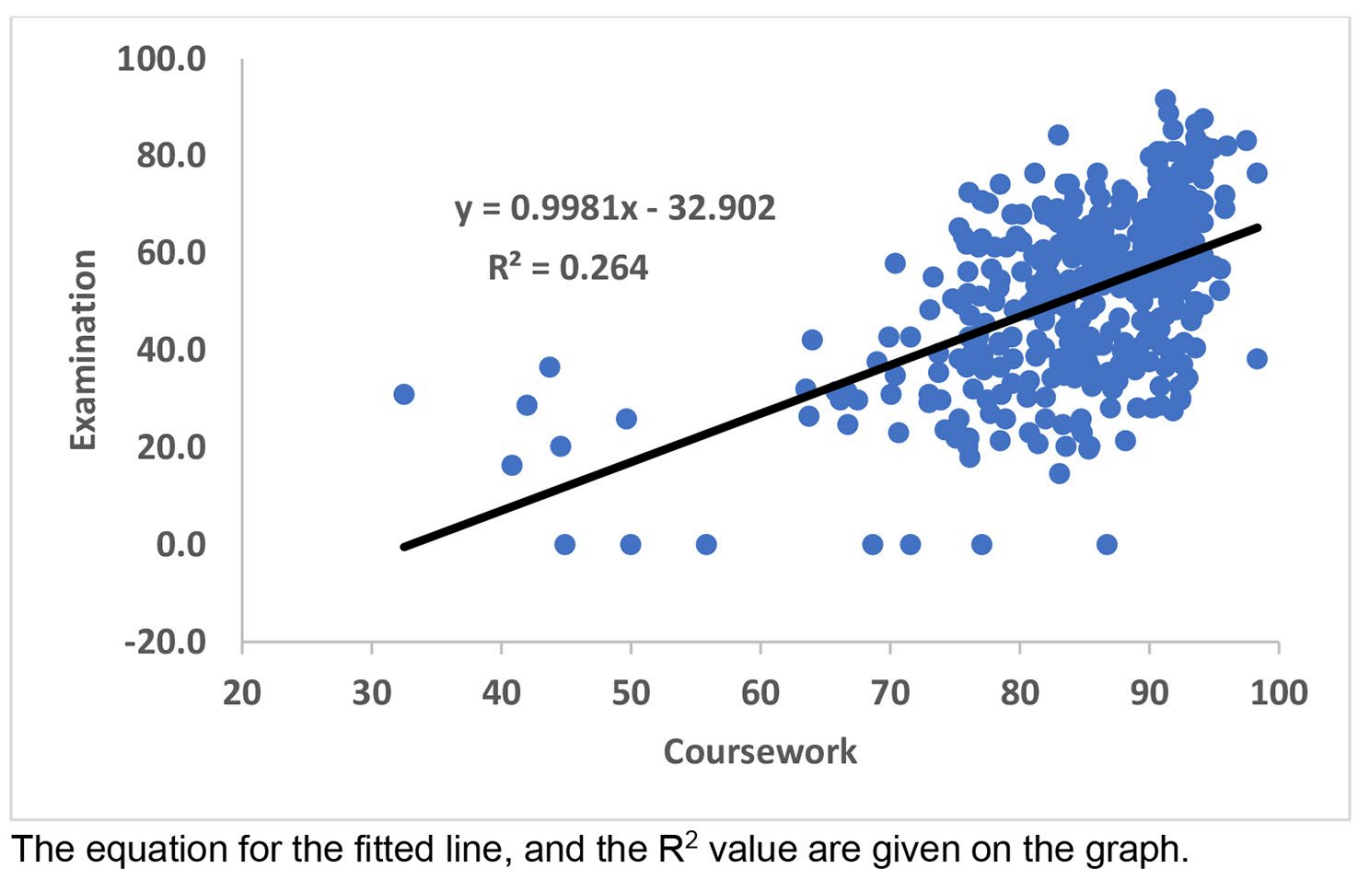
Regression line analysis between the percentage marks in the exam and coursework. The equation for the fitted line, and the R^2^ value are given on the graph

Regression analysis was used to determine whether outcomes in the coursework (combined, laboratory skills or communication in health) predicted the outcome in the exam. In this analysis, the combination of slopes of ~ 1 and R^2^ values of ~ 1 indicate a good correlation. In the analysis of exam mark vs. combined coursework, slopes indicated a good fit, as the values were close to 1. In contrast, R^2^ values did not indicate a good fit (Fig. 1; Table 1), presumably because the marks for coursework were much higher than for the exam. The coefficients demonstrated a moderate correlation between the marks for the exam and the combined coursework (Table 1).

The slopes were lower when the combined coursework was separated into laboratory skills or communication in health vs. the exam, which indicates a lesser fit (Table 1). Pearson’s correlation coefficients indicated that there was moderate correlation between laboratory skills marks, but only a weak correlation between communication in health marks, and the exam marks (Table 1). These findings were not greatly changed by removing the students who (i) did not undertake the exam or (ii) did not sit the exam or undertake a coursework component (Table 2).

### Modelling changing the proportional allocation of marks between coursework and the exam

Reducing the proportion of marks allocated to the exam would have increased the number of students passing the course (Table 3). Due to the high rate in the bioscience course (97.4%), there was only a small ability to increase these rates, and the modelling only resulted in a maximum of 98.4% (Table 3). In contrast, increasing the marks allocated to the exam had the ability to dramatically increase the number of failing students (Table 3). The low failure rate, 2.7%, was increased to a maximum of 42.7% in the modelling (Table 3).

**Table 3:** Actual and modelled data of overall marks, grades, and passing/failing percentages

Data type	% coursework/% exam	Overall mark N = 379	Grade N = 379	Additional students passing^1^ (% passing)	Additional students failing^2^ (% failing)
Actual	60%/40%	71 ± 11	5.3 ± 1.0	(97.4%)	(2.6%)
Modelled	80%/20%	78 ± 9	5.9 ± 0.9	1/10 (97.6%)	
Modelled	100%/0%	84 ± 8	6.4 ± 0.8	5/11 (98.4%)	
Modelled	40%/60%	65 ± 13	4.7 ± 1.1		39/379 (10.3%)
Modelled	20%/80%	58 ± 15	4.1 ± 1.3		96/379 (25.3%)
Modelled	0%/100%	51 ± 18	3.5 ± 1.5		162/379 (42.7%)

1. Additional students passing of the completing students who had failed (% passing of completing students). 2. Additional students failing of the completing students (% failing of completing students). Mark values are the mean ± SD (number of students).

## Discussion

The findings of this study of students of nursing in a bioscience course are like those in a previous study in a different course (pharmacology) [9], despite differences in the types of coursework. Thus, for the completing students (i) marks are higher for coursework than the exam, (ii) there was a moderate association between marks obtained in the exam and coursework, which was moderate for the individual laboratory skills vs. exam but only weak for the group communication in health coursework vs. exam, and (iii) increasing the marks allocated to the exam decreased the number of students who passed the course, whereas decreasing the allocated exam marks increased the number of students passing.

### Marks are higher for coursework than the examination

This is the first study to show that marks for coursework are higher than for the examination for nursing students in a bioscience course. Similar findings have been made previously for students in nursing, paramedicine and optometry undertaking a pharmacology course [9, 16]. The higher marks in coursework may occur for students in nursing regardless of the type of coursework as in this study it was laboratories and communicating in health care, whereas in the pharmacology course the coursework were tutorials and a case-study essay [9, 16]. This needs to be further investigated for other courses for nursing students and in courses for other students.

This difference between marks in examinations and coursework may be due to the examination work representing that of the individual student, whereas the coursework mark may be that of individuals or groups of students. In the present study, the mark for communications in health was a group mark whereas for laboratory skills, the mark was individual. Thus, the performance of weak students, and their marks in communications in health, may be artificially lifted by better students in the group. This problem could be overcome by removing group work from courses. However, there are disadvantages to this, as group work is an important skill for students in many disciplines including nursing. Ways to overcome this ongoing problem is for the marking by academic staff [17, 18] or peers [19] to include marks for individual contributions to overcome the varying contributions by students.

Engagement with a rubic by students has been shown to improve their coursework marks [20]. Thus, it possible that student engagement with a rubric may improve their marks in coursework and explain the higher marks in coursework than examinations. However, to my knowledge, the relationship between the use of a rubric in coursework and final examination marks has not been investigated. A rubric for coursework was not used in the present study or our previous study [8, 16].

### Performance in coursework as a predictor of performance in the exam

The present study showed for students in nursing for bioscience, marks in coursework were a moderate predictor of academic performance in the exam. However, there was a contrast between the types of coursework with the laboratory skills being a moderate predictor, but marks for communications in health being only a weak predictor of academic performance in the exam. Previous studies have shown coursework and its components (tutorials and assignment) to be a weak to moderate predictors (using Pearson’s coefficients) of performance in exams for students in allied health in a pharmacology course [8, 16], and marks in coursework to be a moderate predictor of performance in examinations in a pharmacy course [8]. One feature common to these studies is that coursework involving group work is a poorer predictor of exam performance than individual coursework: communications in health (group) vs. laboratory skills (individual) in present study; tutorials (group) vs. assignment (individual) [9, 16]; the last course in a pharmacy course which had group coursework vs. all the other courses, which had individual coursework [19]. This may be because group work marks are not a good indicator of individual performance, and this needs to be further investigated.

*Altering the marks allocated to the examination changed the number of students who failed or passed.*

With the allocation of marks of 40%/60% to examinations/coursework in the present study, the number of students who failed the bioscience course was very low (2.6%), and consequently it was difficult to increase the passing rate by changing the allocation of marks to coursework. This was confirmed by our modelling showing that the passing rate could only be increased by 1% point. With the 40%/60% allocation, the passing rate was high, 97.4%, and occurred despite 42.7% of students failing the exam component of the course.

The modelling showed that increasing the marks allocated to the exam would have decreased the number of students in nursing who passed the course in bioscience, with 42% failing overall if all the marks had been allocated to the examination. In Australia, the allocation of marks for examination in bioscience courses from nursing programs varies from 15 to 100% (see Introduction). Thus, if there had been more marks allocated to exam in any of these courses where exams marks were less than 100% overall, more students would have failed. Similar findings have recently been reported for modelling coursework and examination marks for students in allied health in a pharmacology course [9, 16].

### Implications of these results

First year students in nursing rapidly lose their recall of bioscience, and less than half, consider they have enough recall to handle further bioscience or pharmacology courses [21]. This situation may have partly arisen from the allocation of marks. The problem is that the students in nursing in the bioscience course, who pass the course based on marks from coursework, but not the examination component, may not have the necessary knowledge to continue their program of study. Thus, the disparity between marks in examinations and coursework needs to be considered. One possible practical solution would be to make it a requirement for the students to pass the examination component to pass the course. This should help but does not guarantee that students have the recall of bioscience necessary for courses later in the program, such as pharmacology, and for any external exams.

### Limitations

The major limitation to this study is that it is of a single course in bioscience, and that some of the results may not relate to other courses being undertaken by students in nursing or other programmes. However, we have previously shown a similar reliance on marks in coursework for the overall success of students in a nursing program undertaking a pharmacology course [9]. Both the bioscience and the pharmacology course were at the same university (QUT), and thus the findings may be limited to this university. In the pharmacology course, we also have evidence that the students in nursing are more reliant on marks from coursework than students in paramedicine or optometry [22]. We do not know whether this finding is limited to students in nursing versus other allied health students or whether it is true for nursing versus other students. Thus, similar analysis needs to be undertaken of other courses to determine whether the findings are specific to science courses for nursing students at one university or can be related to similar science courses at other universities, or to other courses for students in nursing and non-nursing programmes.

## Data Availability

The datasets used and/or analysed during the current study are available from the corresponding author on reasonable request.
